# Quickcard-Based Approach to Guiding Specific Nonpharmacological Treatments in a German Parkinson’s Network

**DOI:** 10.3390/jcm9072272

**Published:** 2020-07-17

**Authors:** Linda Kerkemeyer, Katharina Achtert, Inga Claus, Svenja Happe, Jeannette Overbeck, Nadine Kleen, Anja Palesch, Clara Schmuck, Sabrina Krouß, Jürgen Perick, Luisa Depenbrock, Michael Nagel, Frank Siebecker, Olaf Rose, Tobias Warnecke

**Affiliations:** 1 Institute for Applied Health Services Research (inav), 10117 Berlin, Germany; kerkemeyer@inav-berlin.de (L.K.); achtert@inav-berlin.de (K.A.); 2 Department of Neurology, University of Münster, 48149 Münster, Germany; Inga.Claus@ukmuenster.de; 3 Klinik Maria Frieden Telgte, 48291 Telgte, Germany; Svenja.Happe@Maria-Frieden-Telgte.de; 4 Neurologische Klinik, Christophorus-Kliniken Dülmen GmbH, 48249 Dülmen, Germany; jeannette.overbeck@christophorus-kliniken.de (J.O.); nadine.kleen@christophorus-kliniken.de (N.K.); 5 Fachkräftesicherung im Gesundheitswesen, 46342 Velen, Germany; kontakt@anja-palesch.de; 6 Ergotherapiepraxis Fleischer, 48153 Münster, Germany; clara.schmuck@web.de; 7 Sprachwelt Logopädie, 48431 Rheine, Germany; hallo@sprachwelt-rheine.de; 8 Reha Team Perick GmbH—Ihr Sanitätshaus, 48565 Steinfurt, Germany; j.perick@perick.de; 9 Zentrum für Ambulante Rehabilitation GmbH (ZaR), 48159 Münster, Germany; luisa.depenbrock@zar-ms.de; 10 Klinikum Osnabrück GmbH, 49076 Osnabrück, Germany; michael.nagel@klinikum-os.de; 11 Praxis Neurologie, 48291 Telgte, Germany; fs@neurologie-telgte.de; 12 Impac2t Research, 48147 Muenster, Germany; rose@impac2t.de

**Keywords:** Parkinson’s disease, integrated care network, communication in care networks, nonpharmacological treatment, physiotherapy, occupational therapy, speech therapy

## Abstract

Interdisciplinary care has been shown to be effective at optimizing the treatment of patients with Parkinson’s disease. An optimized collaboration between the various healthcare providers involved in the treatment process facilitates successful care. One of the main shortcomings in the German healthcare system is the limited and unstandardized communication between practitioners. The Parkinson’s network Münsterland+ (PNM+) is an interdisciplinary network of medical and non-medical experts involved in the treatment of Parkinson’s patients: neurologists, physiotherapists, occupational therapists, speech therapists, psychologists, Parkinson’s nurses, pharmacists, patients, and relatives. The PNM+ elaborates guideline-based therapy recommendations, provided as so-called “Quickcards”. Thereby, the communication of the treating neurologist and therapists is based on a coordinated feedback system and suggestions to adequately select and, if necessary, adjust the therapy. In the German healthcare system, with its fragmented structures, the PNM+ and its activities have been shown to enhance integration of the healthcare providers and thereby optimize the care of Parkinson’s disease patients. Future research should evaluate the effects and cost-effectiveness.

## 1. Introduction

Parkinson’s disease (PD) is a neurodegenerative disease with motor and non-motor, e.g., cognitive or gastrointestinal, symptoms [1]. Following dementia, PD is the second most common neurodegenerative disease [2]. Based on a German study conducted in 2015, the prevalence of PD was estimated at 511.4 cases per 100,000 persons. Furthermore, there was an increase in the prevalence of PD with advancing age, peaking in the 80 years age group [3,4]. With increasing life expectancies, a rise in the burden of PD is expected [5].

The disease is referred to as highly complex and remains incurable [6]. Dopamine replacement therapy has been proven to have positive effects on motor functions and thus on quality of life and functional capacity [1]. Apart from drug therapy, clinical practice guidelines (CPGs) recommend nonpharmacological treatments such as physical therapy, occupational therapy, and speech and language therapy [6]. There is growing evidence for the effectiveness of such nonpharmacological treatments offered as a monodisciplinary intervention [7].

Some high-quality international studies have been carried out to assess the effects of physical therapy, occupational therapy, and speech therapy on patients with PD [8,9,10,11,12,13,14,15,16,17]. Some of these studies have been found to have significant positive effects on PD patients [15,16,17]. Firstly, PD patients treated by a specialized physiotherapist had a significantly lower probability of sustaining a Parkinson’s disease-related complication, i.e., hospital admission, than patients treated by a usual care physiotherapist [17]. Secondly, PD patients who received ten weeks of home-based occupational therapy according to practice guidelines showed significantly better self-perceived performance in prioritized activities compared to patients who did not receive any occupational therapy [16]. Thirdly, comparisons between PD patients with various speech treatments and untreated PD patients demonstrated significantly larger improvements in patients who received speech and language therapy [15].

In a cross-sectional study, claim databases of German statutory health insurances showed that, except for physical therapy (36%), less than a third of all Parkinson’s patients received adjunctive therapies such as occupational (6%) and speech therapy (4%). The study confirmed a deficit in nonpharmacological therapies [3]. Moreover, another cross-sectional study analyzed the care situation from the patients’ perspective. On average, patients rated the usage of nonpharmacological therapies as insufficient (scale: from “0 = not at all sufficient” to “5 = absolutely sufficient”), with mean ratings of 2.3 (SD: ± 2.0) for occupational therapy, 2.4 (SD: ± 2.0) for speech therapy, and 3.0 (SD: ± 1.7) for physical therapy [18]. Compared to international standards, the potential of nonpharmacological therapies in Germany has not yet been exploited [17].

A care network presents a potential solution for the mentioned inefficiencies. A network can be defined as a composition of social relations between different actors in the healthcare system. Network relations assume that coordination between actors is based on mutual benefit, reciprocity, and confidence [19]. The treatment within such networks shows potential to better integrate therapies into daily life [18]. Current models of care try to follow multidisciplinary approaches, which are often mistaken for interdisciplinary approaches. The multidisciplinary approach considers practitioners of various disciplines working with the same patient, while approaching the patient from their own perspective and without collaborating with each other. An interdisciplinary approach, however, provides a multidisciplinary team working collaboratively together while trying to include the patient’s perspective [20]. Such integrative forms of care have already been proven successful in the treatment of other chronic conditions. Therefore, network organizations for PD pursue such interdisciplinary treatment approaches [21,22]. The solidarity inside a network carries out positive effects on the treatment coordination. At the same time, a better coordination can lead to lower resource utilization (e.g., fewer hospitalizations) within the healthcare system and higher patient satisfaction [23]. Establishing a network can make a vast contribution toward the optimization of the treatment for PD patients.

A major shortcoming and well-known problem in the German healthcare system for establishing such networks in the outpatient setting is the lack of collaboration between different healthcare providers. This might be related to the gap between in- and outpatient care, as well as to a fragmented structure, resulting in insufficient communication between providers [24]. As a consequence, the prescription of the abovementioned nonpharmacological therapies is usually carried out without consulting the therapist [21,25]. There is no standardized procedure for health professionals communicating with each other, and most of the time, they do so in a limited way [24,26]. Due to the patient’s free choice of physician and therapist, the providers often do not even know each other, which impedes communication. In terms of prescribing nonpharmacological therapies, non-communication between the neurologist and the therapist is an even greater challenge, as the prescription only indicates the underlying disease and not the specific symptoms requiring treatment. As a consequence, the therapist does not generally provide the ideal therapy at the right time [2].

Successful care of PD is calling for optimized interaction between all involved healthcare providers and for the continuous integration of patients and their relatives [5,27,28]. Here, we describe the development of a Quickcard-based approach within an interdisciplinary Parkinson’s network to tackle the deficits in Germany. The aim of the concept is to highlight evidence-based therapy recommendations for pharmacological and especially nonpharmacological treatment in the form of so-called Quickcards. Moreover, these Quickcards will enable standardized interaction during the treatment process to bridge the communication gap between the treating neurologist and therapist. The concept outlines the initial steps along the path of implementing Quickcards into daily routine.

## 2. Methods

### 2.1. Baseline Setting for Quickcard Development

The concept of Quickcards was developed within the context of the Parkinson’s network Münsterland+ (PNM+), which is an existing multidisciplinary network of all medical and non-medical professionals involved in the treatment of people with PD. After an annual preparation phase, PNM+ was officially established in May 2018. So far, the network’s focus has been on the region of Münsterland. Münsterland is a rural area in the north-west of Westphalia in Germany, with a population of approximately 1.6 million inhabitants. The current number of PD patients living in Münsterland is estimated at about 7173 cases [3,29]. PD treatment in Münsterland is provided by an outpatient setting with about 112 neurologists, as well as an inpatient setting with one university hospital, seven acute care clinics, and two rehabilitation clinics [29,30,31].

The existing partner structure is quite heterogeneous, consisting of the following: neurologists working in hospitals and ambulatory sectors, physiotherapists, occupational therapists, speech and language therapists, psychologists, rehabilitation clinics, Parkinson’s disease nurses, Parkinson’s assistants, medical supply stores, and pharmacies. In addition to the professionals, PD patients and their relatives also represent an active part of the network.

The main goal for establishing the network is to provide the best evidence-based care for PD patients. The network was built on the principles of a bottom-up process, meaning that the key components of the network were developed by all network partners. In doing so, the patient was continuously involved in the process. Three predominant key components of the network were set: the first was to establish collaboration between the different providers and settings by promoting intersectoral and interdisciplinary approaches within the network. The second was to further empower the patient by including them in decisions and considering them as a partner. The third was to generate increased knowledge and expertise so that health professionals are supported in approaching and/or referring the patient to a specialized expert within the region.

So far, the network has already been able to realize important key components, with patients being part of the network and working with professionals on an equal footing. The following key components have been successfully established:

1. In terms of promoting collaboration between all partners, the network has created quarterly held multidisciplinary panel meetings as well as a steering committee consisting of the PNM+ initiators and its working groups’ spokesmen. To date, these panel meetings have already taken place 13 times, whereas the steering committee has met five times. Within these meetings, for example, the partners practice skills or discuss cases to further improve their cooperation.
2. Furthermore, the communication and collaboration within the network is enhanced through corporate working groups (WG). Each of these groups discusses and elaborates specific topics related to patient care. So far, the following WGs have been established within PNM+:
  - WG structure of care
  - WG PD and sports
  - WG physical therapy
  - WG relatives
  - WG information/education
  - WG communication and public relations
  - WG psychological aspects
  - WG occupational therapy
  - WG aids and appliances
  - WG innovative technologies
  - WG speech therapy
  - WG medication management
  - WG palliative care
  - WG sleep disorders (and other non-motor symptoms)
3. Lastly, the network has integrated a digital platform which enables the members of PNM+ to work together professionally and flexibly via the internet and communicate easily.

### 2.2. Procedures

The idea for the use of Quickcards is based on the idea that the current prescriptions for nonpharmacological treatments do not depict the underlying symptom in need of treatment. The insufficient nonpharmacological treatments used in the past proved the need for specific therapy references for the therapists. For this reason, the network has decided to establish guideline-based therapy recommendations in the form of so-called ‘Quickcards’. By doing so, the partners’ knowledge and expertise regarding the treatment will be increased. To describe the development process of this novel tool, the Quickcard Dysphagia, dealing with swallowing therapy, was chosen as an example. So far, drafts of other Quickcards have also been developed following the same process: speech therapy, occupational therapy, physical therapy, sleep disorders, and suitability for driving, as well as for aids and appliances.

### 2.3. Process of Quickcard Development

In the first step, the Quickcard Dysphagia was planned by the WG speech therapy consisting of neurologists and speech and language therapists with expertise and experience in treating PD patients. Therefore, pharmacological and nonpharmacological therapy recommendations were established for different symptoms of dysphagia in PD. These recommendations were based on scientific evidence, supplemented with practice-based evidence of the diagnostics and therapy of PD. Therapy recommendations were oriented towards the national evidence-based CPGs for Parkinson’s disease and the Dutch CPG for speech and language therapy in PD [6,32,33]. Additionally, local and regional aspects, such as the availability of specific treatment methods, were taken into consideration.

To allow structured and effective group discussions, nominal group technique methods were applied. The development of the Quickcards was realized in several rounds, where the Quickcards were subjected to an ongoing review process.

The final round took place within the multidisciplinary panel meeting. To test their suitability for daily use, the other PNM+ partners, who also represented the main future users of the Quickcards, received a prototype of the Quickcards in advance to the open round-table discussion within the panel meeting. During the round-table discussion, the PNM+ partners were asked for their feedback in order to identify further potential therapy recommendations. These feedback rounds were performed iteratively until a consensus on the Quickcards and their recommendations was reached.

## 3. Results

The Quickcards illustrate the most important and novel tool for dealing with the mentioned problems regarding communication.

**Figure 1:**
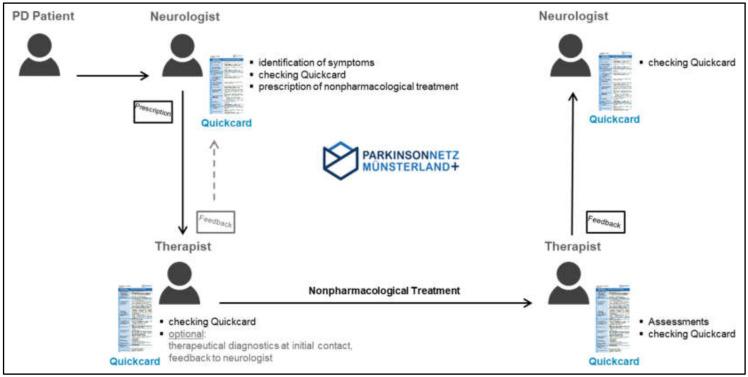
Schematic representation of an interdisciplinary treatment process using Quickcards.

During the consultation, the attending neurologist identifies a symptom of PD, for which he can initiate a nonpharmacological therapy (physiotherapy, occupational therapy, or speech and language therapy) (Figure 1). For example, dysphagia is a common and clinically relevant symptom in PD. Numerous studies on the prevalence of dysphagia in PD have proven that the majority of Parkinson’s patients will report swallowing problems during the course of the disease [34,35]. Accordingly, dysphagia must be addressed by the treating neurologist during consultation with a Parkinson’s patient.

**Figure 2:**
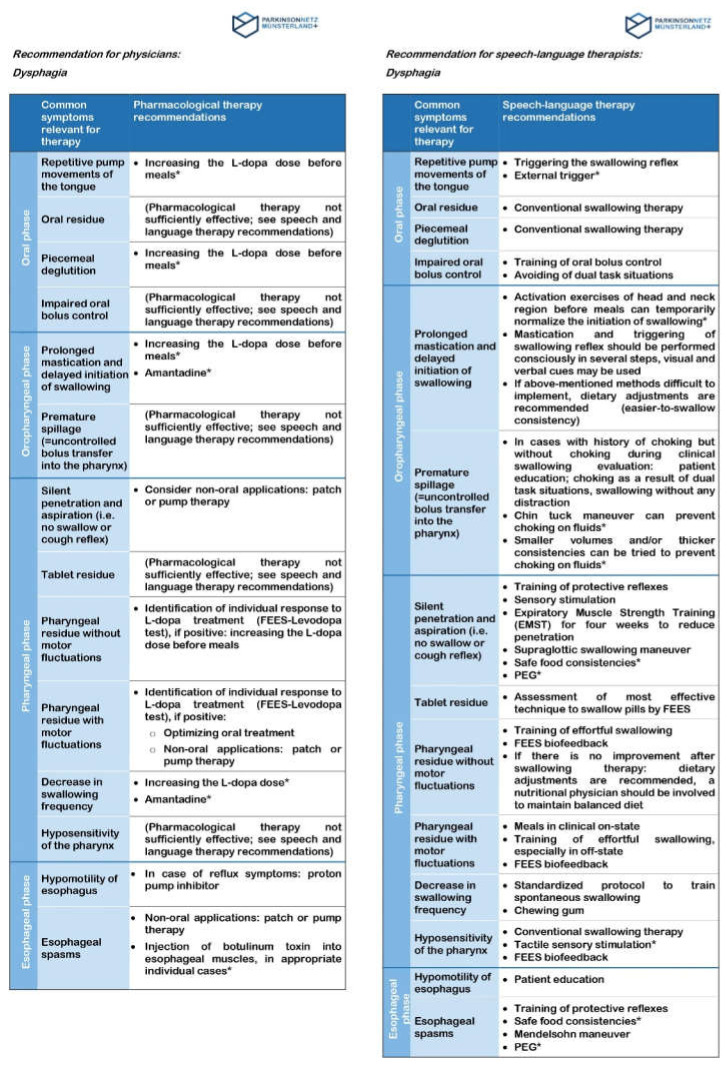
Quickcard Dysphagia. Notes: * Individual assessment of levodopa responsiveness of dysphagia, if positive: consider permanent treatment. Abbreviations: EMST = Expiratory Muscle Strength Training, FEES = fiberoptic endoscopic evaluation of swallowing, PEG = percutaneous endoscopic gastrostomy.

Thus, the Quickcard indicates common symptoms that are relevant for therapy, divided into the oral, oropharyngeal, pharyngeal, and esophageal phases of swallowing. An exemplary presentation of a common symptom of the oral phase would be repetitive pump movements of the tongue. For this symptom, the Quickcard displays therapy recommendations in terms of pharmacological and of speech–language treatments in the corresponding sections. The former consists of an increase in L-dopa doses, while the latter advises an external trigger for the swallowing reflex. The same applies to the remaining phases, with symptoms like prolonged mastication and delayed initiation of swallowing, tablet residue, and hypomotility of esophagus (Figure 2).

The treating neurologist is responsible for anamnesis, clinical diagnostics, and therapy, whereas the speech and language therapist optionally carries out additional therapeutic diagnostics during first contact. For example, the neurologist may identify premature spillage with choking episodes as the most relevant problem of his PD patient with dysphagia. According to the Quickcard, a pharmacological approach is not recommended in this case. For this reason, the treating neurologist prescribes a specific speech–language therapy and documents his recommendation on the Quickcard Dysphagia. The Quickcard will be attached to the prescription and both will be handed out to the patient. During the first therapy session, the speech and language therapist must assess whether the therapist agrees with the treating neurologist’s proposed speech–language therapy recommendations or suggests different therapy procedures. In the example, the Quickcard Dyphagia recommends a training of swallowing without any distraction and the chin tuck maneuver to prevent choking during drinking. Accordingly, the speech and language therapist starts the individually coordinated therapy (= treatment as usual). By doing so, it is ensured that the neurologist’s and therapist’s primary treatment options comply with each other. In case of any disagreement, the neurologist and the therapist must consult with each other. Hence, interdisciplinary communication is a crucial bidirectional component. At the end of the prescribed speech–language therapy sessions, the therapy carried out shows whether the frequency of choking could be reduced during a defined time. Soon afterwards, the speech and language therapist provides coordinated feedback to the treating neurologist by a marking on the Quickcard. In case of success, the therapy can be stopped; otherwise, the speech–language therapist needs to discuss alternative strategies with the neurologist. (Figure 1)

## 4. Discussion

Nonpharmacological therapies for PD are not sufficiently utilized, even though several studies highlight the positive effects of these treatments. One of the most common difficulties that impedes the inclusion of these therapies in daily practice is a suboptimal referral process [36]. Hence, patients in need of a nonpharmacological therapy are often not referred to a therapist at all [36,37].

Furthermore, evidence has been found that being referred to any therapist is not enough. A study conducted within an indication-specific network compared patients treated by a specialized physical therapist and a usual care physical therapist. It was found that being treated by a specialized therapist yielded significantly better outcomes in terms of lower probability of sustaining a PD-related complication than being treated by an usual care therapist [17]. A similar pattern has been found when patients are treated by neurologists who are specialized in movement disorders versus general neurologists [38]. These observations indicate that there is a need for specialization in terms of chronic and complex diseases like PD [39].

These findings indicate that it is crucial to not only enable referrals to nonpharmacological treatments but to also provide neurologists and therapists with CPG-based therapy recommendations when being confronted with the need for a nonpharmacological treatment for a specific symptom.

The Netherlands was one of the first countries to focus on a similar topic with the implementation of ParkinsonNet and can therefore be considered a role model: ParkinsonNet is a network that tackles a multidisciplinary approach, patient-orientation, and coordinated care. Hereby, its main components are evidence-based treatment guidelines, a focus on nonpharmacological therapies, constant education of participating network partners, support of neurologists in referrals to further therapists, and optimized communication via a platform [22].

The German healthcare system is characterized by highly fragmented processes and funding mechanisms [24,26]. In a healthcare system with such fragmented structures, it is crucial to further promote the integration of all healthcare providers of a PD patient in order to create the right context for innovations such as the implementation of the Quickcards. It has been shown that the creation and implementation of a network for a specific disease can facilitate and stimulate collaboration between healthcare providers. A few years ago, the necessity to change the current structures in the German healthcare system towards an optimized and specialized outpatient care of PD patients has been stated. Since then, steps towards a specialized care have been taken by establishing interdisciplinary treatment approaches in the form of network organizations like the PNM+.

In this paper, it was demonstrated that the network has already implemented important activities to achieve its main goals, namely to establish collaboration between the different providers and segments, to promote patient empowerment, and to generate increased knowledge and expertise. Furthermore, the specialization of therapists within the care of PD patients was shown to be of the utmost importance. With the existence of different medical disciplines and derived medical specialists, the question of establishing specialization within a therapist’s education can be raised. In doing so, therapists can choose their specialization of interest while in education and later offer optimized care for complex diseases like PD.

The Quickcards provide neurologists and therapists with clear standards for referring and treating patients according to their current and specific symptoms, rather than providing the patients with unspecific nonpharmacological treatments. In training courses offered to therapists, the handling of Quickcards and the proper exercise of the portrayed nonpharmacological recommendations will be taught, since most of these exercises are not part of the therapist’s standard procedures.

Early experiences in day-to-day practice showed that various therapy options—in both pharmacological and nonpharmacological therapy—are becoming increasingly transparent for the treating neurologist or, rather, the therapist. The development and initial implementation process of Quickcards generated an increase in knowledge on both sides. Consequently, the structured exchange and cooperation on which Quickcards focus help to tackle difficulties in the bilateral communication between neurologist and therapist. During a pilot test phase within the PNM+, uncertainties regarding the handling and transfer of Quickcards arose. However, PNM+ partners, as well as non-PNM+ healthcare providers, were enthusiastic about the idea and were creative when transferring the Quickcards, e.g., by attaching Quickcards to medical or therapy reports.

Quickcards, with their bidirectional standards, try to find novel ways to overcome barriers in the German healthcare system. By referring the patient to a specialized expert and tailoring the therapy to the patient’s needs, the quality of care can most likely excel standard care.

However, some limitations of the described approach must be acknowledged: (1) Quickcards focus on only one part of the communication gap in the treatment of PD. At present, only medical and non-medical providers with direct involvement in the nonpharmacological treatment process of a PD patient are integrated into the coordinated communication. In the future, it will be necessary to ensure its broad-scale implementation. The opening-up of PNM+ and the greater penetration of existing structures are essential for the integration and the subsequent long-term use of Quickcards in treatment as usual. (2) Currently, Quickcards only represent paper-based prototypes that need further development, such as their digitalization. In addition, the digital version of Quickcards should be linked to the electronic patient record in the future. (3) The outlined PNM+ is to be considered as a regional solution designed for the needs of the specific population. Such networks can take many different forms and are highly dependent on the underlying population and regional structures. The potential of integrated care approaches for PD is widespread internationally. Recently, a systematic review has found various approaches of integrated care projects for PD [40]. The recommendations regarding future integrated care approaches given in this review comply with the principles of the Quickcards. Therefore, the Quickcard approach could be transferred and adapted according to the specific needs and structures of other regions.

After all, existing research could prove that the use of a multidisciplinary team consisting of medical and non-medical providers working together collaboratively is inevitable in the treatment of PD. Further research is needed to evaluate the effects and the cost-effectiveness of the Quickcards.
